# Fifteen years of continuous improvement of quality care of type 2 diabetes mellitus in primary care in Catalonia, Spain

**DOI:** 10.1111/j.1742-1241.2011.02872.x

**Published:** 2012-02-16

**Authors:** M Mata-Cases, P Roura-Olmeda, M Berengué-Iglesias, M Birulés-Pons, X Mundet-Tuduri, J Franch-Nadal, B Benito-Badorrey, J F Cano-Pérez, on behalf of the Diabetes Study Group in Primary Health Care (GEDAPS: Grup d'Estudi de la Diabetis a l'Atenció Primà ria de Salut, Catalonian Society of Family and Community Medicine)

**Affiliations:** 1 Primary Care Center (PCC) La Mina, Sant Adrià de BesòsBarcelona, Spain; 2 Barcelona Ciutat Research Support Unit – IDIAP Jordi GolredIAPP, Barcelona, Spain; 3 PCC Badía del VallésBarcelona, Spain; 4 PCC Florida Nord, L’Hospitalet de LlobregatBarcelona, Spain; 5 PCC PoblenouBarcelona, Spain; 6 PCC El CarmelBarcelona, Spain; 7 PCC RavalBarcelona, Spain; 8 Servicio de Endocrinologia, Hospital del MarBarcelona, Spain

## Abstract

**Aims:**

To assess the evolution of type 2 diabetes mellitus (T2DM) quality indicators in primary care centers (PCC) as part of the Group for the Study of Diabetes in Primary Care (GEDAPS) Continuous Quality Improvement (GCQI) programme in Catalonia.

**Methods:**

Sequential cross-sectional studies were performed during 1993–2007. Process and outcome indicators in random samples of patients from each centre were collected. The results of each evaluation were returned to each centre to encourage the implementation of correcting interventions. Sixty-four different educational activities were performed during the study period with the participation of 2041 professionals.

**Results:**

Clinical records of 23,501 patients were evaluated. A significant improvement was observed in the determination of some annual process indicators: HbA_1c_ (51.7% vs. 88.9%); total cholesterol (75.9% vs. 90.9%); albuminuria screening (33.9% vs. 59.4%) and foot examination (48.9% vs. 64.2%). The intermediate outcome indicators also showed significant improvements: glycemic control [HbA_1c_ ≤ 7% (< 57 mmol/mol); (41.5% vs. 64.2%)]; total cholesterol [≤ 200 mg/dl (5.17 mmol/l); (25.5% vs. 65.6%)]; blood pressure [≤ 140/90 mmHg; (45.4% vs. 66.1%)]. In addition, a significant improvement in some final outcome indicators such as prevalence of foot ulcers (7.6% vs. 2.6%); amputations (1.9% vs. 0.6%) and retinopathy (18.8% vs. 8.6%) was observed.

**Conclusions:**

Although those changes should not be strictly attributed to the GCQI programme, significant improvements in some process indicators, parameters of control and complications were observed in a network of primary care centres in Catalonia.

**What′s known**

The results of clinical studies have shown that implementation of intervention programmes for the management of type 2 diabetes mellitus has a positive impact in quality of care. However, limited data are currently available from primary care settings.

**What′s new**

The present study describes the impact of the Group for the Study of Diabetes in Primary Care intervention programme on type 2 diabetes mellitus quality of care in primary care settings in Spain by analysis of the trend of quality indicators.

## Introduction

The benefits of controlling type 2 diabetes mellitus (DM) and the associated cardiovascular risk factors are well established and reflected in the current clinical practice guidelines (1–4). However, the results of several cross-sectional studies have highlighted the difficulties in achieving the goals as well as the full implementation of the clinical recommendations (5–9). The results of consecutive cross-sectional observational studies have shown some positive trends on both process indicators and degree of disease control (10–17).

Moreover, the results of several clinical trials conducted to evaluate different quality improvement programmes at both primary and secondary care centres have shown significant improvements in both process and intermediate outcome indicators (degree of glycemic control and other risk factors) with some impact on final outcome indicators like hospital admissions and health-related costs (18–20). The feedback of the indicators′ results to the health providers is considered the basis for any quality improvement intervention (21–23). In Spain, there is limited information published in this regard, mainly from cross-sectional studies (5–9,17).

In 1992, the Group for the Study of Diabetes in Primary Care (GEDAPS) was founded by the Catalan Society of Community and Family Medicine to implement the aims of the Saint Vincent Declaration (1). In 1993, the group published the first edition of the ‘Guidelines for Diabetes Management in Primary Health Care in Spain’ that included both clinical and organisational recommendations and also defined a set of quality care indicators. The guidelines were updated in the following years (1995, 1998, 2000 and 2004) (2). In parallel, the group developed the GEDAPS continuous quality improvement (GCQI) computer programme to facilitate clinical audits. The programme constructed automatically process and outcome indicators based on the data recorded by the participant centres from random samples of patients′ medical records. In 1996, the programme was expanded to other Spanish regions (17). The GCQI programme was mainly based on the feedback of the results of the clinical indicators to the participating centres to promote interventions to improve quality of care.

In 1993, the first evaluation of quality of care of type 2 DM in primary care settings took place in Catalonia. The evaluation was repeated in 1995, 1998, 2000, 2002 and 2007. At the same time, as part of the intervention, a series of workshops and seminars were launched to publicise and implement the GEDAPS guidelines as well as the recommendations to improve early detection of the disease, treatment, management of diabetes complications and specific workshops to analyse quality indicators and propose local interventions to improve patient′s quality of care.

The aim of the present study was to describe the impact of the GEDAPS intervention programme on type 2 DM quality of care in primary care settings, by analysing the trend of quality indicators collected in assessments that took place between 1993 and 2007 in Catalonia, Spain.

## Research design and methods

### Study design

The GCQI programme gathered information from primary care centres (PCC) on process and outcomes indicators in a sample of their patients. To promote their participation, letters by ordinary mail and electronic mails (years 2000–2002) were sent to all PCC in Catalonia. The planned 2005 survey was not conducted because the medical records were being computerised during the previous years. During the last evaluation (2007), several investigator meetings around the territory were conducted to encourage participation in the study, regardless of their participation in the previous evaluations and to present changes in data entry using a webpage (http://www.redgdps.org/).

Health providers entered patient data using the GCQI computer programme that immediately provided the results of a set of disease-specific processes and outcomes indicators. Data were subsequently sent by disk (1993–2000), electronic mail (2002) or introduced directly in the redgedaps.org web (only in 2007). The GCQI computerised programme was specifically designed to perform periodic evaluations (audits) in primary care centres. The programme was based in two principles: data collection from each participant centre (each participant centre had a nurse or physician responsible of the survey) and subsequent data feedback to the centres. Thus, each centre was able to compare their data during subsequent assessments (internal comparison) and with data from other centres (external comparison). The gold standard for each indicator in each evaluation was the overall result of all the participating centres. Each centre then compared their own results with the global results (gold standard) to find differences that required to be improved.

Health providers were instructed to obtain a random sample from the medical records of type 2 DM patients with a follow-up greater than 6 months since diagnosis. A total sample of five patients multiplied for the number of basic care units (physician/nurse), with a minimum of 30 patients per centre, was required. A preselection of medical records with an additional 20% was performed. In those cases that did not fulfil the inclusion criteria the medical record was replaced by the next one of the same gender. Exclusion criteria included: type 1 DM; follow-up exclusively by an endocrinologist and short life expectancy (terminal patients or those that received home care).

Because of the retrospective nature of the study, based only on clinical records, patients were not required to give written informed consent. To assure anonymity, data were collected and recorded using two different files: one included demographic variables and the other one included clinical variables linked by a consecutive record number. The study design and the GCQI programme were presented and approved by the Consell Assessor de la Diabetis (Advisory Board on Diabetes) of the Health Department of the Autonomous Government in Catalunya that behaved as Institutional Review Board.

Data were collected from paper medical records from 1997 to 2002 and from electronic records in 2007. Data about the characteristic of the centre (rural or urban), number of doctors and nurses team (basic care units), total population and prevalence of diabetes were fulfilled by the professional responsible of the evaluation.

### GCQI programme interventions

The GCQI programme was mainly based on the feedback of the results of clinical indicators that were sent after each evaluation to the participating centres to promote interventions to improve quality of care. On the other hand, as part of the intervention programme, 55 courses, seminars and workshops were conducted during the study period to disseminate the GEDAPS Guidelines and its recommendations, and a total of 2041 health professionals (physicians and nurses) attended. The main aim of the courses and workshops was to encourage the global management of the disease, not only to improve glycemic control but also to promote the proper management of other cardiovascular risk factors as well as the performance of annual activities leading to early detection and treatment of diabetes complications. In relation to the nurses clinical activities, a special emphasis was put on reviewing the educational interventions, annual screening activities and the degree of disease control in each patient, and not be limited to explain diet or performing clinic measurements, that is the traditional role of nurses in our country.

Moreover, after the evaluations that took place in 1995 and 1998, nine decentralised workshops with the participation of 289 health professionals from 151 primary care teams (43% of the primary care centres of Catalonia), were conducted to analyse the results and identify healthcare difficulties to propose local corrective interventions.

### Variables

#### Demographic and clinical characteristics

Age; gender; weight; height; body mass index (BMI), blood pressure; glycated haemoglobin (HbA_1c_); total cholesterol and HDL-cholesterol; year of diabetes diagnosis; number of doctor or nurse visits, number of educational interventions recorded per year; antidiabetic treatment and smoking status.

#### Process and outcome indicators of quality of care

The following indicators, that have been previously described elsewhere, were studied (17): Process indicators: (i) related to the organisation: No visit related to diabetes recorded; less than three nursing visits; less than three educational interventions of different topic (whatever the number of visits required to perform the intervention for each topic); practice of self-monitoring blood glucose; (ii) laboratory measurements: at least one HbA_1c_ determination; two or more HbA_1c_ determinations; at least one total cholesterol determination; at least one HDL-cholesterol determination; at least one microalbuminuria screening determination; (iii) physical examinations: weight measurements (three or more times a year); funduscopy done by an ophthalmologist; foot examination; Outcome indicators: (i) intermediate outcomes: Good glycemic control (HbA_1c_ ≤ 7% or 57 mmol/mol); acceptable glycemic control (HbA_1c_ ≤ 8% or 68 mmol/mol); very poor glycemic control (HbA_1c_ > 10% or 89 mmol/mol); HDL-Cholesterol > 40 mg/dl (1.03 mmol/l); total cholesterol ≤ 250 mg/dl(6.47 mmol/l) (acceptable control); total cholesterol ≤ 200 mg/dl (5.17 mmol/l) (strict control); BMI < 30 kg/m^2^; BP ≤ 140/90 mmHg (acceptable control); BP ≤ 130/80 mmHg (strict control); active smoking; (ii) final outcomes: diabetic foot (ulcers + amputations); diabetic foot ulcers; amputations of lower limbs; nephropathy (microalbuminuria or macroalbuminuria); retinopathy; amaurosis; coronary artery disease (including angina); stroke (including transient ischaemic attack); hospital admissions because of amputation, hypoglycemia or any other reason, but with plasma blood glucose > 500 mg/dl(27.28 mmol/l).

### Statistical considerations

Continuous variables were described using the mean and standard deviation. Categorical variables are described as percentage with the confidence interval of 95% (95% CI). The SPSS.11 statistical program was used for all statistical analyses.

## Results

During the study period (1993–2007) 55 seminars were conducted and a total of 2041 health professionals (physicians and nurses) from 1084 centres attended. Table 1 summarises the characteristics of the primary care participant centres. The PCC covered one-third of the population of Catalonia (7,364,068 individuals in 2007). The number of participant centres increased over time, from 1993 to 2002, with a decline during the last evaluation (2007). More than half of the centres were urban, reaching 67.3% in 2007. The prevalence of type 2 DM increased over time, from 3.3% in 1993 to 5.4% in 2007 (relative increase of 63%).

**Table 1:** Participant centres and patient characteristics in each evaluation*

	1993	1995	1998	2000	2002	2007
**Characteristics of participant centres**						
Number of participating centres	57	75	75	78	96	52
Urban centres (%)	54.4 (41.1–66.9)	56 (44.8–67.2)	56.6 (45.4–67.8)	52.6 (41.5–63.7)	57.3 (47.4–67.2)	67.3 (54.5–80.0)
Number of basic care units (physician + nurse)	433	565	609	680	846	637
Total assigned population	954,126	1,251,689	1,367,639	1,474,242	1,888,593	1,126,532
Assigned population over 14 years old	694,450	982,567	1,058,903	1,203,310	1,541,618	938,429
Number of patients with diabetes over 14 years	22,663	38,697	51,776	63,831	83,859	55,350
Prevalence of diabetes in patients over 14 years (%)	3.3 (3.0–3.5)	4.0 (3.8–4.2)	4.9 (4.7–5.1)	5.3 (5.1–5.5)	5.4 (5.2–5.5)	5.4 (5.2–5.6)
**Patients' characteristics**						
Number of participants	2239	3532	4217	4564	5819	3130
Gender (% female)	56.6 (54.5–58.6)	54.5 (52.9–56.1)	52.9 (51.4–54.4)	52.1 (50.6–53.5)	51.8 (50.5–53.1)	48.5 (46.7–50.2)
Age (years), mean (SD)	65.2 (10.2)	66.3 (10.3)	67.2 (10.6)	67.1 (10.8)	67.3 (10.9)	68 (11.7)
> 65 years old patients (%)	50.9 (48.8–53.0)	55.4 (53.8–57.0)	59.6 (58.1–68.1)	60.0 (58.6–61.4)	60.5 (59.2–61.8)	60.2 (58.5–61.9)
Diabetes duration (years), mean (SD)	7.5 (7.1)	7.8 (7.5)	8.2 (7.1)	7.6 (6.8)	8.0 (6.9)	7 (5.6)
Prevalence of obesity (BMI ≥ 30 kg/m^2^) (%)	37.0 (35.0–39.0)	37.0 (35.4–38.6)	39.2 (37.7–40.7)	40.5 (39.1–41.2)	42.6 (41.3–43.9)	50.3 (48.5–52.0)
HbA_1c_ (%), mean (SD)	7.7 (1.9)	7.6 (1.6)	7.1 (1.6)	7.0 (1.7)	7.1 (1.4)	6.8 (1.4)
Physician visits related to diabetes, mean (SD)	3.7 (3.4)	2.9 (3.7)	2.7 (2.7)	2.8 (2.7)	2.6 (2.4)	4.1 (4.0)
Nurse visits related to diabetes, mean (SD)	5.1 (3.7)	5.1 (4.2)	4.6 (3.3)	4.2 (3.2)	3.6 (2.6)	4.8 (4.1)
**Antidiabetic treatment (%)**						
Diet and exercise alone	25.7 (23.9–27.5)	27.7 (26.2–29.2)	29.4 (28.0–30.8)	27.9 (26.6–29.2)	25.4 (24.3–26.5)	22.3 (20.8–23.7)
Oral antidiabetic drugs	52.2 (50.1–54.3)	50.0 (48.3–51.2)	49.9 (48.4–51.4)	51.7 (50.2–53.1)	54.6 (53.3–55.9)	60.5 (58.8–62.2)
Insulin (monotherapy)	20.0 (18.3–21.7)	20.2 (19.9–21.5)	17.8 (16.6–18.9)	15.2 (14.2–16.2)	12.3 (11.5–13.1)	7.3 (6.4–8.2)
Insulin + oral antidiabetic drug	2.1 (1.5–2.7)	2.0 (1.5–2.5)	2.8 (2.3–3.3)	5.2 (4.6–5.8)	7.6 (6.9–8.3)	10.0 (8.9–11.0)

* Data expressed as absolute numbers, means (standard deviation, SD) or percentages (95% confident interval)

### Patients′ characteristics

The clinical records of 23,501 patients were evaluated. Table 1 summarises the characteristics of patients in each evaluation. Mean age increased from 65.2 years (SD: 10.2; range: 30–93) in 1993 to 67 years (SD: 10.9; range: 31–99) in 2007, with a significant progressive increase in the percentage of patients > 65 years old (50.9% vs. 60.2%). Other significant differences found between 1993 and 2007 evaluation included: lower number of female patients (56.6% vs. 48.5%); higher prevalence of obesity (37% vs. 50%); shorter time of diabetes duration (7.5 years vs. 7 years) and lower HbA_1c_ concentration (7.7% or 64 mmol/mol vs. 6.8% or 55 mmol/mol).

Throughout the study, more than half of the participants received oral antidiabetic treatment, whereas approximately 20% of the patients received insulin (alone or in combination therapy). Among this latter patient population, the percentage that received combined treatment (insulin + oral antidiabetics) increased significantly over time (2.1% vs. 10%) (Table 1).

### Process indicators

#### Related to organisation

Throughout the study the number of patients that did not have any diabetes-related visit recorded significantly decreased (5.1% in 1993 vs. 2% in 2007), with an increase in the percentage of patients that visited the nurse more than three times per year (27.3% in 1993 vs. 31.5% in 2007). Doctors and nurses visits tend to decrease progressively, but increased in 2007. Likewise, a significant decrease was observed in the percentage of patients receiving less than three different annual educational interventions (74.6% in 1993 vs. 58.3% in 2007) (Table 2).

**Table 2:** Evolution of process and outcome indicators*

	1993 (*n* = 2239)	1995 (*n* = 3532)	1998 (*n* = 4217)	2000 (*n* = 4567)	2002 (*n* = 5819)	2007 (*n* = 3130)	Difference
**Process indicators**							
Related to the organisation							
No diabetes-related visit recorded	5.1 (4.2–6.0)	3.0 (2.4–3.6)	1.7 (1.3–2.1)	2.1 (1.7–2.5)	2.2 (1.8–2.6)	2.0 (1.5–2.5)	−3.1 (−4.1 to −2.0)
Less than three nursing visits	27.3 (25.5–29.2)	27.6 (26.1–29.1)	28.4 (27.0–29.8)	32.8 (31.4–34.2)	35.9 (34.7–37.1)	31.5 (29.9–33.1)	+4.2 (1.8 to 6.7)
Less than three educational interventions	74.6 (72.8–76.4)	56.3 (54.7–57.9)	61.2 (59.7–62.7)	67.2 (65.8–68.6)	64.6 (63.4–65.8)	58.3 (56.6–60.0)	−16.3 (−18.8 to −13.8)
Control parameters							
At least one blood pressure measurement	94.5 (93.6–95.4)	93.0 (92.2–93.8)	93.9 (93.2–94.6)	92 (91.2–92.8)	92.2 (91.5–92.9)	92.3 (91.4–93.2)	−2.2 (−3.5 to −0.9)
At least one HbA_1c_ measurement	51.7 (49.6–53.4)	70.2 (68.7–71.7)	77.6 (76.3–78.9)	82.8 (81.7–83.9)	85.3 (84.4–86.2)	88.9 (87.8–90.0)	+37.2 (34.9 to 39.5)
Two or more HbA_1c_ measurements	30.0 (28.1–31.9)	41.1 (39.5–42.7)	40.6 (39.1–42.1)	42.2 (40.8–43.6)	55.5 (54.2–56.8)	40.4 (38.7–42.1)	+10.4 (7.8 to13.0)
At least one total cholesterol measurement	75.9 (74.1–77.7)	80.5 (79.2–81.8)	83.1 (82.0–84.2)	84.4 (83.4–85.5)	86.5 (85.6–87.4)	90.9 (89.9–91.9)	+15.0 (13.0 to 17.1)
Weight control (three or more times a year)	44.9 (42.8–47.0)	32.7 (31.2–34.3)	31.3 (29.9–32.7)	33.5 (32.1–34.9)	40.5 (39.2–41.8)	40.2 (38.5–41.9)	−4.7 (−7.4 to −2.0)
Screening for complications							
Funduscopy performed by an ophthalmologist	52.2 (50.1–54.3)	48.4 (46.8–50.1)	52.6 (51.1–54.1)	52.2 (50.8–53.7)	54.3 (53.0–55.6)	49.0 (47.3–50.8)	−3.2 (−5.9 to −0.5)
Foot examination	48.9 (46.8–51.0)	58.3 (56.7–59.9)	54.3 (52.8–55.8)	54.1 (52.7–55.6)	56.6 (55.3–57.9)	64.2 (62.5–65.9)	+15.3 (12.6–17.9)
Determination of microalbuminuria	33.9 (31.9–35.9)	49.0 (47.4–50.7)	62.5 (61.0–64.0)	68.7 (67.4–70.0)	72.8 (71.7–73.9)	59.4 (57.7–61.1)	+25.5 (23.6–27.4)
**Outcome indicators**							
Intermediate outcomes							
Good glycemic control (HbA_1c_ ≤ 7%) (57 mmol/mol)	41.5 (39.5–43.5)	42.2 (40.6–43.8)	54.7 (53.2–56.2)	58.7 (57.3–60.1)	56.5 (55.2–57.8)	64.2 (62.5–65.9)	+22.7 (20.1 to 25.3)
Acceptable glycemic control (HbA_1c_ ≤ 8%) (68 mmol/mol)	62.6 (60.6–64.6)	65.4 (63.8–67.0)	74.0 (72.7–75.3)	77.6 (76.4–78.8)	78.6 (77.6–79.7)	83.3 (82.0–84.6)	+20.7 (18.3 to 23.1)
Very poor glycemic control (HbA_1c_ ≥ 10%) (89 mmol/mol)	13.4 (12.0–14.8)	10.4 (9.4–11.4)	5.7 (5.0–6.4)	5.7 (5.0–6.4)	4.6 (4.1–5.1)	4.2 (3.5–4.9)	−9.2 (−10.8 to −7.6)
HDL-cholesterol > 40 mg/dl (1.03 mmol/l)	74.7 (72.9–76.5)	72.2 (70.7–73.7)	77.2 (75.9–78.5)	78.0 (76.8–79.2)	77.6 (76.5–78.7)	83.0 (81.7–84.3)	+8.3 (6.5 to10.5)
Total cholesterol ≤ 250 mg/dl (6.47 mmol/l)	73.1 (71.3–74.9)	77.0 (75.6–78.4)	77.4 (76.1–78.7)	85.8 (84.8–86.8)	87.0 (86.1–87.9)	92.4 (91.5–93.3)	+19.3 (17.2 to 21.3)
Total cholesterol ≤ 200 mg/dl (5.17 mmol/l).	25.5 (23.7–27.3)	29.4 (27.9–30.9)	31.9 (30.5–33.3)	41.3 (39.9–42.7)	46.3 (45.0–47.6)	65.6 (63.9–67.3)	+ 40.1 (37.6 to 42.5)
Body mass index < 30 kg/m^2^	63.0 (61.0–65.0)	63.0 (61.4–64.6)	60.8 (59.3–62.3)	56.9 (55.5–58.3)	57.4 (56.1–58.7)	49.7 (48.0–51.5)	−13.3 (−16.0 to −10.6)
BP ≤ 140/90 mmHg	45.4 (43.3–47.5)	47.4 (45.8–49.1)	50.1 (48.6–51.6)	54.9 (53.5–56.3)	58.8 (57.5–60.1)	66.1 (64.4–67,8)	+20.7 (18.0–23.3)
BP ≤ 130/80 mmHg	22.0 (20,3–23,7)	23.3 (21.9–24.7)	24.6 (23.3–25.9)	26.8 (25.5–28.1)	30.5 (29.3–31.7)	35.0 (33.3–36.7)	+13 (10.6–15.4)
Active smoking	13.4 (12.0–14.8)	14.3 (13.2–15.5)	15.0 (13.9–16.1)	14.4 (13.4–15.4)	15.4 (14.5–16.3)	13.6 (12.4–14.8)	−0.2 (−2.1 to 1.6)
Final outcomes (prevalence on complications)							
Diabetic foot (ulcers plus amputations)	9.5 (8.3–10.7)	6.0 (5.2–6.8)	4.2 (3.6–4.8)	3.5 (3.0–4.0)	3.0 (2.6–3.4)	3.2 (2.6–3.8)	−6.3 (−7.7 to −5.0)
Diabetic foot ulcers	7.6 (6.5–8.7)	5.4 (4.7–6.2)	3.4 (2.8–4.0)	2.7 (2.2–3.2)	2.3 (1.9–2.7)	2.6 (2.0–3.2)	−5 (−6.8 to −3.2)
Amputations of lower limbs	1.9 (1.3–2.4)	1.6 (1.2–2.0)	0.8 (0.5–1.1)	0.8 (0.5–1,1)	0.7 (0.5–0.9)	0.6 (0,3–0,9)	−1.3 (−1.9 to −0.7)
Nephropathy (micro or macroalbuminuria)	7.1 (6.0–8.2)	6.7 (5.9–7.5)	7.1 (6.3–7.9)	7.0 (6.3–7.7)	7.1 (6.4–7.8)	9.9 (8.8–11.0)	+2.8 (1.3 to 4.3)
Retinopathy	18.8 (17.2–20.4)	14.5 (13.3–15.7)	13.5 (12.5–14.5)	10.3 (9.4–11.2)	9.8 (9.0–10.6)	8.6 (7.6–9.6)	−10.2 (−12.1 to −8.3)
Amaurosis	2.7 (2.0–3.4)	3.3 (2.7–3.9)	3.1 (2.6–3.6)	2.1 (1.7–2.5)	2.1 (1.7–2.5)	0.3 (0.1–0.5)	−2.4 (−3.1 to 1.7)
Coronary artery disease	12.9 (11.5–14.3)	12.0 (10.9–13.1)	12.5 (11.5–13.5)	11.2 (10.3–12.1)	12.5 (11.7–13.4)	11.3 (10.2–12.4)	−1.6 (−3.4 to 0.2)
Stroke	6.8 (5.8–7.8)	6.8 (6.0–7.6)	6.6 (5.9–7.4)	5.9 (5.2–6.6)	5.7 (5.1–6.3)	6.3 (5.5–7.2)	−0.5 (−1.8 to 0.9)
Hospital admission for amputation, hypoglycemia or glycemia > 500 mg/dl (27.76 mmol/l)	3.8 (3.0–4.6)	4.9 (4.2–5.6)	6.3 (5.6–7.0)	7.6 (6.8–8.4)	6.8 (6.2–7.5)	6.8 (5.9–7.7)	+3.0 (1.8 to 4.2)

* All results are expressed as percentage with 95% confident interval related to the population of the year evaluated.

#### Control parameters

**Figure 1:**
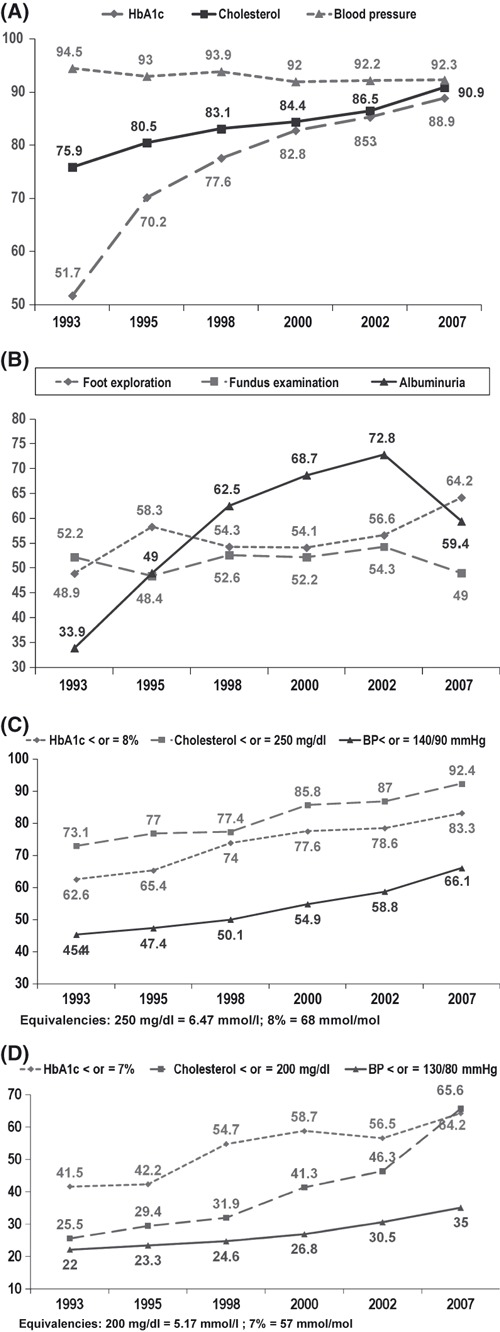
(A) Process indicators: HbA_1c_, total cholesterol and blood pressure. Percentage of patients with at least one annual measurement. (B) Process indicators: complications screening. Percentage of patients with annual foot exploration, fundus examination and albuminuria screening. (C) Intermediate outcome indicators: percentage of patients with acceptable control of HbA_1c_, total cholesterol and blood pressure. Equivalencies: 250mg/dl = 6.47 mmol/l; 8% = 68 mmol/mol. (D) Intermediate outcome indicators: percentage of patients with strict control of HbA_1c_, total cholesterol and blood pressure. Equivalencies: 200mg/dl = 5.17 mmol/l; 7% = 57 mmol/mol

A significant increase in the number of annual analytical determinations of HbA_1c_ (51.7% in 1993 vs. 88.9% in 2007) and total cholesterol (75.9% vs. 90.0%) was observed (Table 2 and Figure 1A).

#### Complications screening

As for regular checkups, there has been improvement in the percentage of patients that have been tested for microalbuminuria (33.9 in 1993 vs. 72.8% in 2002), although a fall was observed in 2007 (59.4%). The funduscopy examination initially improved, but then remained stable with some downward trend in the last assessment (52.2% vs. 49%). In addition, foot exploration increased significantly throughout the study (48.9% vs. 64.2%) (Table 2).

### Outcome indicators

#### Trends in intermediate outcome indicators

Throughout the study significant improvements were observed in glycemic control, increasing the percentage of patients with HbA_1c_ ≤ 8% or 68 mmol/mol (from 62.6% to 92% and reducing the number of patients with very poor glycemic control (HbA_1c_ ≥ 10% or 89 mmol/mol) from 13.4% to 4.2%. In addition, a significant increase in the percentages of patients with acceptable control of total cholesterol (≤ 250 mg/dl-6.47 mmol/l), (from 73% to 92.4%) and blood pressure control (≤ 140/90 mmHg) (from 45.4% to 57.1%) was also noted (Table 2 and Figure 1B). Using more strict control criteria, HbA_1c_ ≤ 7% or 57 mmol/mol increased from 41.5% to 64.2%, total cholesterol (≤ 200 mg/dl-5.17 mmol/l) from 25.5% to 65.6% and blood pressure (≤ 130/80 mmHg) from 22% to 35% (Table 1 and Figure 1C). In contrast, no change in the percentage of active smokers (13.4% and 13.6%) and an increase in obese patients (BMI ≥ 30) (from 37% to 50%) were noted (Table 2).

#### Trends in final outcome indicators

There has been a significant decrease in the prevalence of retinopathy (from 19.8% to 8.6%) and a slight increase in the prevalence of nephropathy (micro or microalbuminuria, from 7.1% to 9.9%) (Table 2). Prevalence of diabetic foot ulcers (from 7.6% to 2.6%) and amputation (from 1.9% to 0.6%) has also significantly decreased. In contrast, reductions in macrovascular complications have been much poorer: ischaemic heart disease (12.9% vs. 11.3%) and stroke (6.8% vs. 6.3%). The number of patients that required hospital admission because of hyperglycemic decompensation increased significantly throughout the study (from 3.8% to 6.8%).

## Discussion

The present study analyses the evolution of type 2 DM management in primary care settings in Catalonia. During the study period, a significant increase in the prevalence of DM2 and obesity was observed, probably related to the epidemic increase in the prevalence of obesity in the western countries during the last decades. However, there were no important changes in the mean age and sex distribution, duration of the disease and steps of treatment.

The significant improvements observed in some of the process indicators, in particular glycemic control, blood pressure and cholesterol, may have contributed to the reduction of key chronic complications associated with the disease, such as retinopathy and diabetic foot. These improvements meet the expectations of reducing the percentage of complications included among the goals of the Declaration of Saint Vincent (1).

The analysis of the evolution of process indicators highlight the improvement of laboratory measurements (HbA_1c_, cholesterol, and albuminuria) that are essential to assess the need or the effect of treatments as well as patient risk. However, the limited improvement observed in foot and funduscopy examinations noted in the study should be carefully analysed because such explorations are essential to early detection.

The improvements in type 2 DM process indicators observed in the present study are comparable with the trends described in other studies. Thus, the results of a population-based study that compared the results of successive cross-sections in 1988 (1024 patients) and 2006 (13,078 patients) to assess changes in quality of type 2 DM care in United States by using standardised measures showed a significant improvement in HbA_1c_ (from 34% to 51%), funduscopy (from 63.2% to 67.7%) and foot examination (from 65.4% to 68.3%) (10). Similarly, in another study conducted among Medicare beneficiaries with diabetes between 1992 (150,000 patients) and 2001 (230,000 patients) the number of HbA_1c_ and funduscopy examinations significantly increased (from 31% to 76% and from 49% to 57% respectively) (11). Other studies conducted in the UK, Israel, Netherlands, Sweden and U.S. also show improvements in the indicator trends although such studies had limitations regarding the number of patients analysed (reduced sample sizes) and length of follow-ups (< 5 years) (13–16). The effect of pay-for performance on the quality of primary care has been recently evaluated in England (16).

The improvement in all composite measures of quality (80% and 90% in the determination of HbA_1c_, blood pressure and lipids) confirmed the benefits of such strategy. In addition, between 1998 and 2007 foot exploration increased from 57.4% to 91.5% and funduscopy examination from 69.4% to 81.1%. Such increases were significantly higher than those observed in our study.

With regard to intermediate outcomes in the British intervention, the proportion of patients who achieved the target A_1C_ value (≤ 7.5%) increased from 59.1% to 66.7%, the proportion that achieved the target BP (≤ 145/85 mmHg) increased from 70.9% to 80.2%, and finally, the proportion that achieved the target TC value (≤ 5 mmol/l) increased from 72.6% to 83.6%^16^. Although the differences in the targets between the British intervention and our study does not allow a head-to-head comparison of the results; nevertheless both showed a similar positive trend.

In the Spanish health system the role of nurses in the management of type 2 DM has increased steadily over the past 20 years. Nurses often perform, in addition to educational endeavours, foot examination as well as analytical determinations and funduscopy examination requests. Therefore, it is important to highlight their potential role in the improvements obtained in the present study. In fact, different experiences in the U.S. have shown that nurses can achieve equal or better results compared with physicians, especially when are provided with software tools to help decision-making (24–26).

Concerning the changes observed in the present study with regard to intermediate and final outcome indicators should not be attributable solely to the GCQI programme, but instead, are a reflection of the progressive changes in type 2 DM disease management experienced by our health system. This time trend of improvement in diabetic control has also been observed in prior cross-sectional studies conducted in US (10,11) and Europe (12,14,15). Moreover, an improvement in outcome indicators has also been described in a prior study conducted in the US. Thus, medical records from Medicare patients, analysed between 1992 (150,000 patients) and 2001 (230,000 patients) showed a reduction of foot amputations in 22% associated with a 4% increase in the prevalence of retinopathy (11).

The significant reduction observed in diabetic foot lesions and retinopathy may reflect the educational and prevention interventions conducted by the health professionals. However, these improvements may also be due in part to the intensification of type 2 DM diagnosis screenings, the lowering of the glycemic cut-offs in the 1997 diagnostic criteria from 140 to 126 mg/dl (7.77–7 mmol/l) and the improvement in diagnosis registration. Such changes have led to the inclusion of patients in earlier stages of the disease and therefore increasing the percentage of patients belonging to the less severe category. This could explain the fact that the HbA_1c_ percentage and the prevalence of microvascular complications or time since diagnosis have decreased in recent assessments. However, the prevalence of heart disease and stroke has not decreased and this could be because of the similar mean age of patients in each evaluation and the limited impact of glycemic control on macrovascular complications (27). Finally, one unexpected result is the slight increase observed in hospital admissions that could be explained by an improvement in clinical records, but we cannot exclude an increase in severe hypoglycemia because of the intensification of pharmacological treatment. As the indicator only includes the number of admissions, but not the reason, it is impossible to discard the possible effect of the feedback of the results on glycemic control that could lead to an overtreatment of some patients. However, as the results about glycemic control are from the whole centre and not at individual level (nor doctor neither patient) it seems improbably that the feedback could affect directly their patients. At the same time, the threshold for the intensification of treatment in our GEDAPS guidelines and in the pay-for-performance of our institution was 8% and this relatively soft threshold could protect our patients from overtreatment. The registration of the number of severe hypoglycemic episodes would be a very interesting indicator to add to future audits.

The main limitations of this study include the design of the study, based on quality interventions rather than epidemiological or investigational purposes, and the lack of a control group. Because of the voluntary participation of the centres and the length of the study it has been impossible to recruit a group of centres from other areas or regions acting as a control group. In relation to the validity of collected data, studies of quality improvement are based on the principle that all unregistered activity is considered not being made. Taking into account the overloaded conditions of working in primary care it can be assumed that health professionals were not able to perform a comprehensive record of the activities, especially in educational issues. In the study period, the computerised medical record was generalised throughout primary care in Catalonia from 2003 to 2004, therefore almost all the medical records reviewed until 2002 were handwritten. In contrast, in the 2007 assessment results were collected from electronic medical records, which may explain the increased number of visits or foot examinations registered in the last assessment. However, no improvements in funduscopy examination or nephropathy screening were observed. As for any study of continuous quality improvement programme, it should not be ruled out that the observed improvements are merely a reflection of an upgrading in medical record registration (23,28). However, some studies suggest that improvements in electronic management system are not always accompanied by improvements in health outcomes (20).

Another possible limitation of the study is that participation was voluntary, therefore it could be hypothesised that only more motivated centres for diabetes control participated in the assessments. However, the fact that the health provider responsible for data reviewing was motivated did not preclude that the remaining professionals of the PCC were motivated for diabetes management.

Finally, we must raise the question of whether improvement in process indicators involves improvements in health outcomes indicators. Most interventions show an improvement of the process indicators and intermediate outcomes (21–23,28). The comprehensive registration of activities does not guarantee a strict clinical attitude and therefore treatment modification or intensification could not be associated with achievement of treatment goals. However, there is consensus that process indicators are the only tools to monitor the impact of quality interventions because final outcome indicators are neither considered sensitive nor specific as quality of care measures (28).

It can be concluded that during the study period there have been improvements in the registry of health activities as well as performance of physical examinations and laboratory tests. The improvements achieved in glycemic control and other risk factors may have contributed to the reduction in foot amputations and diabetic retinopathy observed. Although those changes should not be attributed strictly to the GCQI programme, they reflect an improvement in the health of type 2 DM patients managed in primary care in our country.

## Appendix 1: List of participating investigators in the GEDAPS evaluations

Mª Victoria Abellan, Mª Teresa Adell; Raquel Adoer; Mª Eugenia Adzet; Carina Aguilar; Emilia Alabau; Inmaculada Alegre; Mercé Algans; Herminia Algilaga; Mercedes Aliaga; Rosa Alie; Josep Alins; Carmen Almirall; Josep Almirall; Amelia Alonso; Otilia Alonso; Tomas Alonso; Judit Alsina; Ana María Altaba; Isabel Alvarez; Mercedes Alvarez; Mercé Alvarez; Francesc Anguera; Gloria Antón; Charo Añaños; J. Ignacio Aoiz; Enric Aragonés; Eugenia Arasa; Mª Josefa Arasa; Teresa Areny; Teresa Arnau; Enric Arroyo; Carmen Asensio; Mercé Aubanell; Francisco Avila; Teresa Aviño; Pilar Babi; María Badenes; Rosa Maria Badia; Pilar Baillo; Josep Lluía Ballvé; Carmen Baquer; Elena Barnat; Mª Carmen Barrero; Joan Barrot; Rosa Ana Bas; Núria Bastida; Enric Bayona; Domenech Benaigues; Mª Rosa Benedicto; Belén Benito; Carmina Bentue; María Berengué; Marta Berga; Francisco Berlanga; Martí Birules; Alba Blanch; Mª Isabel Bobé; Miriam Boira; Neus Bonamusa; Neus Boqué; Eulalia Borrell; Manel Borrell; Montserrat Bosch; Concepción Bou; Consol Bou; Javier Buil; Magda Bundo; Esther Caballé; Isabel Caballero; Mª Carmen Caballero; Josep Caballero; Rosa Caballol; Teresa Cabases; Juan Josep Cabré; Mª Angels Calaf; Glora Mª Calleja; Maria Luisa Calvet; Ramón Camps; Rosa Canals; Silvia Canivell; Juan Francisco Cano; Josep Cañellas; Dolors Capdevilla; Anna Carabi; Carmen Carretero; Carolina Carrillo; Ricart Carillo; Mª Angels Casals; Jordi Casanovas; Nieves Casbas; Pilar Casellas; Mª José Castany; Mercé Castaño; Mª Jose Castelar; Carme Castelló; Joan Castells; Dolors Catalá; Matilde Catalá; Mª Jesús Cerain; Encarnación Checa; Ester Chiveches; Carmen Ciria; A. Claramunt; Purificación Claver; Joan Clotet; Yolanda Coccor; Fina Coll; Josep Coll; Montserrat Coma; Vicent Coma; Josefina Comas; Joan Conxat; Ferran Cordon; Carmen Coronado; Carme Cortina; Francesc Xavier Cos; Pilar Coscullera; Engracia Costa; Joan Costa; Xavier Costa; Jose Mª Cots; Ramon Creus; Inés Cruz, Lourdes Cruz; Nuria Culi; Montserrat Dalmau, Josep Davins; M. Teresa de Cos; Josep Anton de la Fuente; Nuria de la Iglesia; Mercé de la Torre; Juan A de Luna; Lourdes de Marcos; Rosa Mar de Miguel; Marta de Puig; Marta del Moral; Carmen Delgado; Francisca Delgado; Cecilia Domenech; José María Domingo; María Isabel Domingo; Roser Dot; Carme Echevarria; Pilar Enseñat, Mª Luisa Escandón; Mercé Escarrá; Alex Escosa; Lídia Escur; Jordi Espinás; Montserrat Espuga; Teresa Esquerra; Marià Esquerrá; Josefa Estany; Montserrat Estruch; Sion Fabregat; Antonia Fargas; Assumpta Farrás, Pere Farrás; Montserrat Farrús; Eugeni Fau;Montserrat Fernandez; Carmen Fernández; Maria Rosario Fernández; Pablo Fernández; Mª Carmen Fernández; Judit Ferragutcasas; Maria Ferré; Daniel Ferrer-Vidal; Xavier Figueras, Xavier Flor; Carme Florensa; Pere M.Flores; Quintí Foguet; Mar Foix; Pilar Font; Jordi Forcada; Maria Carme Forn; Josep Franch; Rosa Freixedes; Miquel Fuentes; José Mª Fuste; Pilar Fusté; Carmen Galera; Gisela Galindo; Roser Galitó; Anna Mª Garcia; Eva Garcia; Isabel Garcia; J. Manel Garcia; Laura Garcia; Vega Garcia; Gracia Garcia; Concepció Garcia; M. Teresa Garcia; Jose Maria Garrido; Anna Gasol; M. Amparo Gaytano; Dolors Gelabert; Jordi Gentille; Francesc German; Faustino Gerri; Rosa M Gimbert; Virginia Golobart; Josep Gomà; Albert Gomez; Alicia Gonzalez; Clementina Gonzalez; J. Carles Gonzalez; Matilde Gonzalez; Tamara Gonzalez; Maria Gonzalez; Mª Rosa Gorgot; Lluís Gracia; Imma Grau; Neus Gregori; Montserrat Grivé; Teresa Gros; Elisenda Guarné; Asunción Guarner; Faustino Guerri; Jesús Domingo Guevara; Mª Antònia Gutíerrez; María Cruz Guzmán; Anna Rosa Hernandez; Mª Mercè Hernandez; Enric Hernández; Anna Hernández; Juan Herreros; Beatriz Hortangas; Monica Ibañez; Carme Iglesias; Pedro Iglesias; Pascual Jaime; Ricardo Jara; Rosa Juanola; Joan Juvanteny; Eduard Kronfly; Cristina Laserna; Carmen Lecumberri; Xavier Lecumberri; Montserrat Ledesma; M. José Ledó; Mª Antonia Llauger; Josep Lluís Llor; Judit Llussà; Flora Lopez; Regina Lopez; Angels López; Anna López; M.José Lorente; Gustavo Losada; Joan Lozano; Marissa Madrid; Carme Mallorqui; Sara Marcelo; Yolanda Marcos; M. Victoria Marina; Carmen Marquilles; Jordi Marti; Laia Martí; Raquel Martín; José Antonio Martin; Ana Martinez; Daniel Martinez; Dolores Martinez; Dolors Martinez; Ignacio Martinez; Miguel Martinez; Carmen Martinez; Rafael Martinez; Valenti Martinez; Sara Martinez de Arenzana, Raquel Martos; Rosa Mª Masdeu; Anna Masseda; Josep Massons; Manel Mata; Enriqueta Matheu, Anna Mayoral; Laura Mayordomo; Pilar Medina; Salvador Medina, Cèlia Melèndez; Ana Menal; Mª Angeles Méndez; Miquel A. Mercadè; Josep Mercader; Xavier Mestre; Concepción Mestres; Rosa Mar Miguel; Anna Miralbés; Mercè Miranda; Sònia Miravet; Magda Mitjavila; Àngels Mollói Josep Felip Monclus; Susanna Montesinos; Javier Monteverde; Sandra Moraleda; Carlos Moreno; Maria de la Sierra Moreno; Miguel Moreno; Rocío Moreno; Xavier Mundet; Pere Munt; Aser Muñoz; Roser Muñoz; Marife Muñoz; Rosa Blanca Muñoz; Josep Murria; Josep Mussoll; Carme Nabau; Carmel Nadal; Antonia Navarro; Juan Navarro; Maria del Mar Navarro; Pilar Navarro; Cristina Nieto; Vicente Nieto; Pere Noe; Ana M.Nogales; Concepcion Nogués; Marisol Oliva; Irene Oliva; Mercedes Oliver;Mª Angels Oliveras; Miquel Oller; Montserrat Ortigas; Jacinto Ortiz; Jose Osvet; Isabel Otzet; Antonio Padilla; Jesús Pages; Elisenda Palet; Merce Pallarés; M. Pilar Pallarés; Mª Teresa Pallerols; Nuria Palou; Clara Pareja; Rosa Pascual; Maria Pastoret; M. Florencia Patittucci; David Pedrico; Magda Pedrosa, Margarida Pelegri; Francisca Peñas; Josep Maria Pepió; Consol Peracaula; Julio Perez; Elena Pérez; Miguel Peso;Jordi Pi; Magda Pi; Antoni Plana; Isabel Plaza; Montserrat Policarpio; Isabel Porta; Montserrat Portas; Joan Prat; Paloma Prats; Pilar Prellezo; Neus Profitós; Francesc Puig; Josep Puig; Marta Puig; Xavier Puigdengolas; Montserrat Pujiiula; Ramon Pujol; Dolors Pujol; Francisca Puntes; Núria Rabentós; Joana Ramon; Ana Maria Ramos; Estibaliz Redondo; David Riba; Fidel Riba Barrés; Enriqueta Ribas; Ricart Ribas; Teresa Ribé; Trinitat Ribes; Carme Riera; Natàlia Riera; José Luís Rivero; Rosabel Roca; Antonio Rodriguez; Francisco Rodriguez; Javier Rodriguez; Mònica Rodríguez; Olivia Roig; Montserrat Romaguera; Xavier Romani; Eva Romero; Mª Dolors Romero; Esther Ros; Pilar Roura; Montserrat Roures; Aurora Rovira; Carles Rubio; Laura Rubio; Montserrat Rubio; Irene Ruiz; M.Paz Ruiz; Miriam Ruiz; Joana Ruiz; Teresa Sabartes; Primitivo Sabaté; Maria A.Sagarra; Josep M Sagrera; Isabel Sales; Anneliese Salzer; Angela Sanchez; Rosalia Sanchez; Yolanda Sanchez; Carme Sánchez; Josep M.Sánchez; Consol Sánchez; Enric Sanchis; Teresa Sangra; Laura Sanguesa; Maria Sanmartin; Neus Saun; Meritxell Saura; Montserrat Saus; Carme Sebastià; Eva Segura; Amparo Segura; Gemma Sellas; Beatriz Sena; Mª José Sender; Dolores Serra; Marta Serra; Imma Serrabasa; Lidón Serrano; Vanesa Serrano; Maria Serratosa; Angels Sieria; Silvia Sierra; Josep Mª Sierra; Josefina Sirvent; José Francisco Sobrino; Silvia Solà; Pascual Solans; Mª Carmen Soldado; Júlia Solé; Montserrat Solé; Magda Solé; Merce Soler; Luís Solsona; Mª Teresa Sopena; Marta Sorribes; Edith Steiner; Ana Mª Suarez; Esperanza Suros; Robert Surriba; Pau Surribes; M. Luz Talavera; Sol Taramon; Maria Victoria Tarin; Salvador Tarradas; Eduard Tarrago; Luisa Tarrida; Joan Tobias Pedro Tomás; Marta Torne; Anabel Torras; Emma Torres; Natividad Torres; Josep Ubach; Antonio Ubieto; Sara Unanue; Luís Carlos Valladares; María José. Vendrell; Cristina Verdera; Maria Vernet; Raquel Vicente; Roser Vicente; Antonieta Vidal; Mª Antonia Vila; Carme Vila; Marga Vilageliu; Luisa Vilaginer; Josep Vilalta; Pere Vilalta; Eulàlia Vilaplana; Esther Viler; Lidia Villagrasa; Merce Villaro; Jordi Villegas; Antoni Vives; Rosa Vives; Nuria Xifro; Rosa Elena Yañez; Montserrat Zamora
